# Sidonia and Nicu de Barcsy: a famous mother with post-partum hirsutism after giving birth to a famous son with idiopathic short stature

**DOI:** 10.1530/EDM-23-0095

**Published:** 2024-08-27

**Authors:** Wouter W de Herder

**Affiliations:** 1 Department of Internal Medicine, Sector of Endocrinology, Erasmus MC, Dr. Molewaterplein40, Rotterdam, the Netherlands

**Keywords:** Adult, Paediatric, Female, Male, White, Hungary, United States, Ovaries, Pituitary, Gynaecological endocrinology, Error in diagnosis/pitfalls and caveats, August, 2024

## Abstract

**Summary:**

At the end of the 19th century, an 18-year-old lady gave birth to a well-proportioned, though very small, son. After delivery, the mother developed a full-grown beard, whereas the son always remained of small stature. The mother developed diabetes mellitus and died, aged 59, from a complicated severe cold. The son died at the age of 91 because of chronic kidney disease. The differential diagnosis in the son is isolated growth hormone deficiency. The mother might have suffered luteoma of pregnancy, polycystic ovary syndrome (PCOS), or Sertoli–Leydig cell tumor(s). The two cases are apparently coincidental/not related in pathophysiology.

**Learning points:**

- Hirsutism occurring directly postpartum can have several causes.
- Patients with isolated growth hormone deficiency can live a long life without the substitution of growth hormone.
- Coincidence does not necessarily imply correlation.
- In the past, patients with endocrine disorders like severe hirsutism or small stature were employed at circuses and fairs to entertain the audience as curiosities.

## Background

Two different relatively rare endocrine disorders in a mother and son apparently seem to suggest some kind of pathophysiologic relationship. However, the presented cases, postpartum hirsutism and small stature/isolated growth hormone deficiency, seem to be unrelated and coincidental. Despite the fact that both disorders occurred in an era when endocrine testing was not possible yet, the clinical deduction can come close to probable differential diagnoses.

The appreciation of endocrine disorders leading to particular phenotyping (bearded female, small stature male) by the general public in the pre-World-War-II era was quite different from nowadays: patients were generally not seen as patients but as show objects, or curiosities. The impression, moreover, is that the bearded female patient was treated with dignity.

## Case presentation and outcome

**Figure 1:**
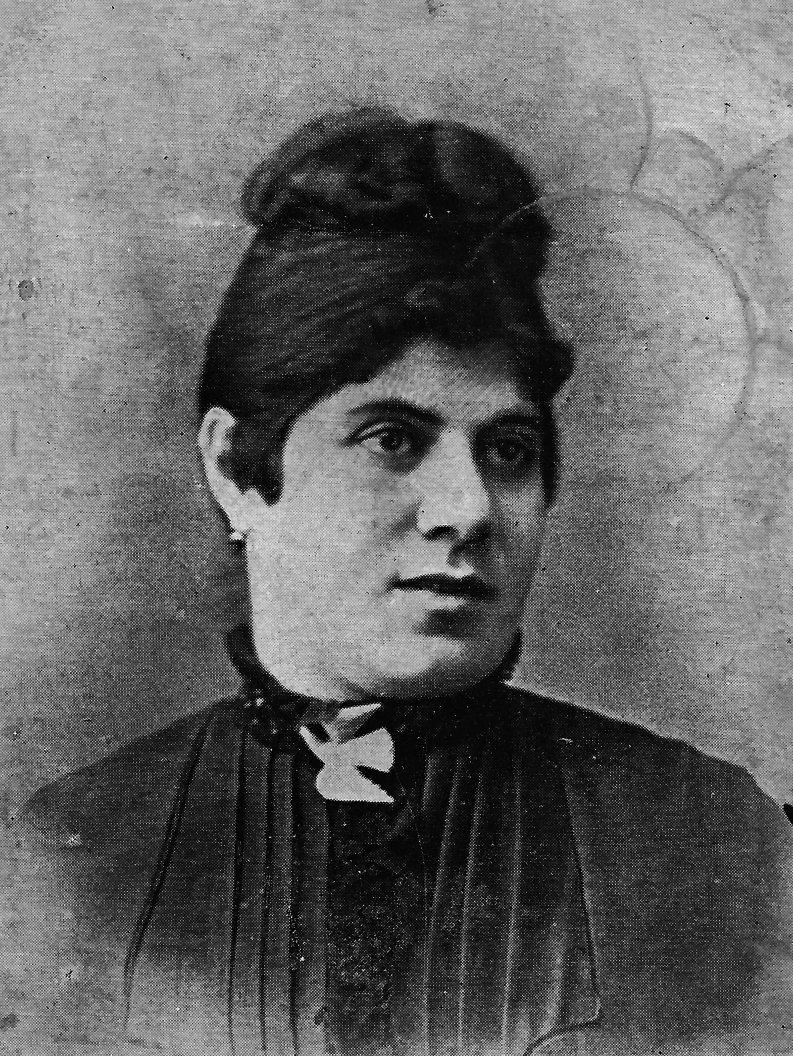
Sidonia de Barcsy (1 May 1866 – 19 October 1925) before she developed a beard. Picture from a picture postcard published by Mucke & Schaerf in Gera-Reuβ, Thüringen, Germany. Postcard in the collection of WW de Herder.

**Figure 2:**
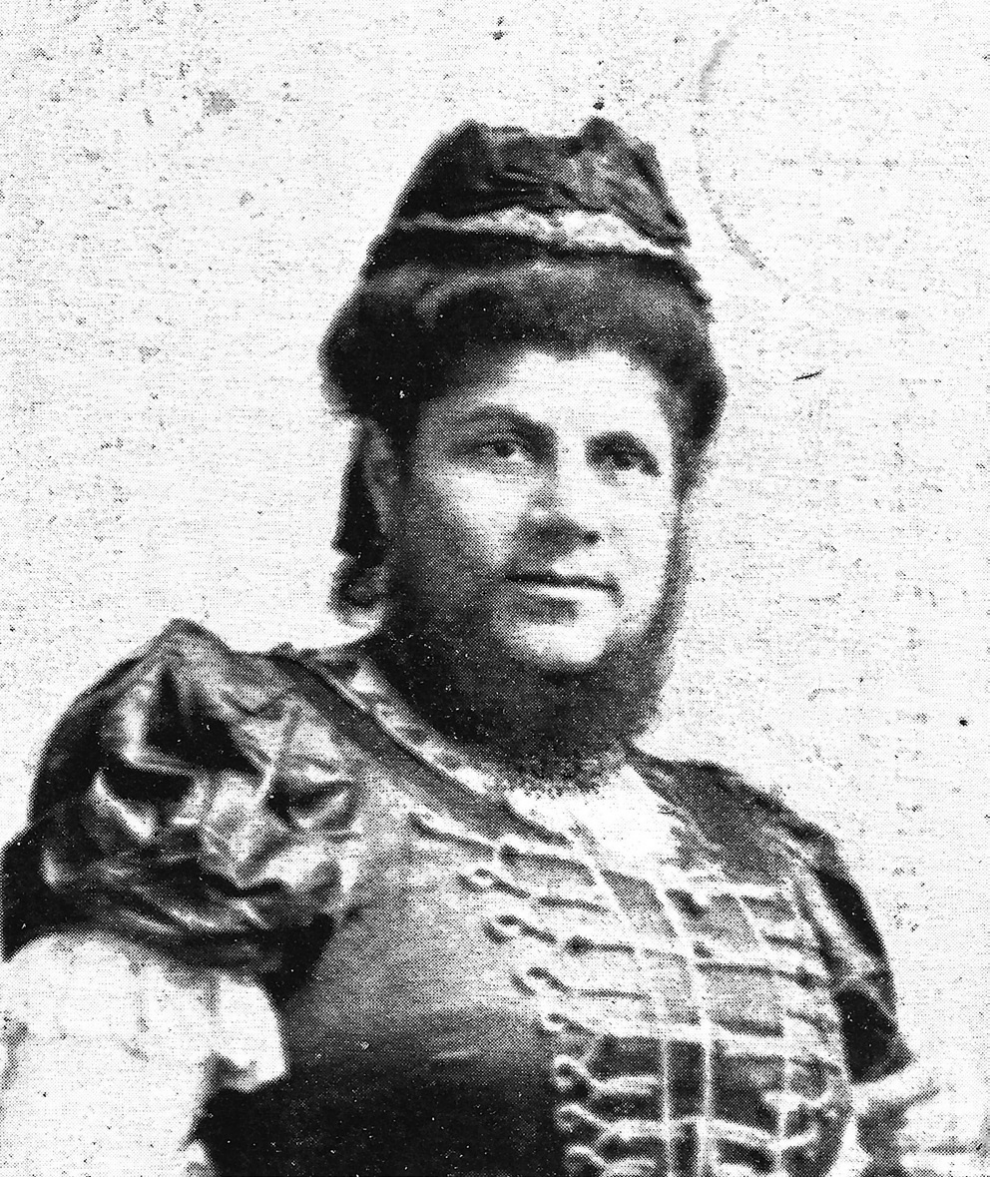
Sidonia de Barcsy (1 May 1866 – 19 October 1925) after having developed a full-grown beard. Picture from a picture postcard published by Mucke & Schaerf in Gera-Reuβ, Thüringen, Germany. Postcard in the collection of WW de Herder.

**Figure 3:**
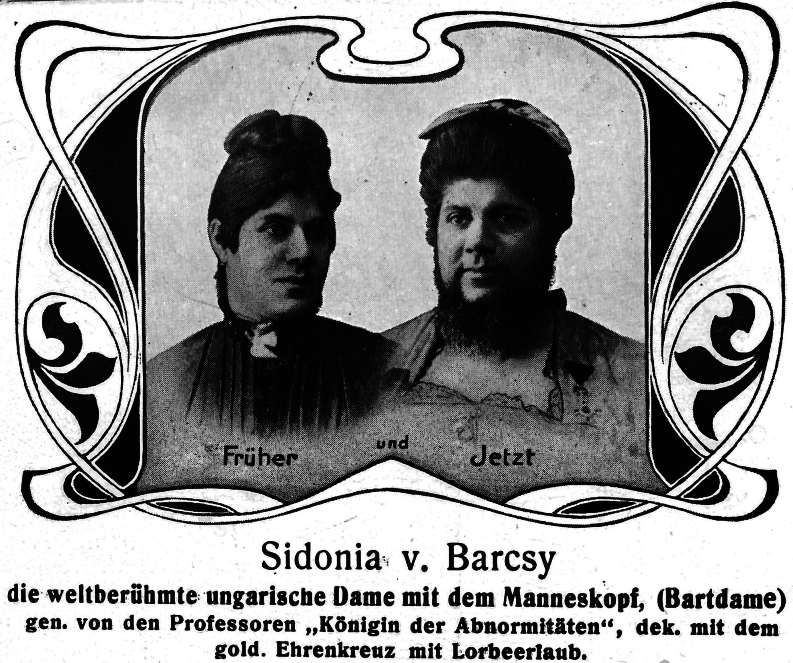
Sidonia de Barcsy (1 May 1866 – 19 October 1925) before and after having developed a full-grown beard. Fragment from a picture postcard. Postcard in the collection of WW de Herder.

**Figure 4:**
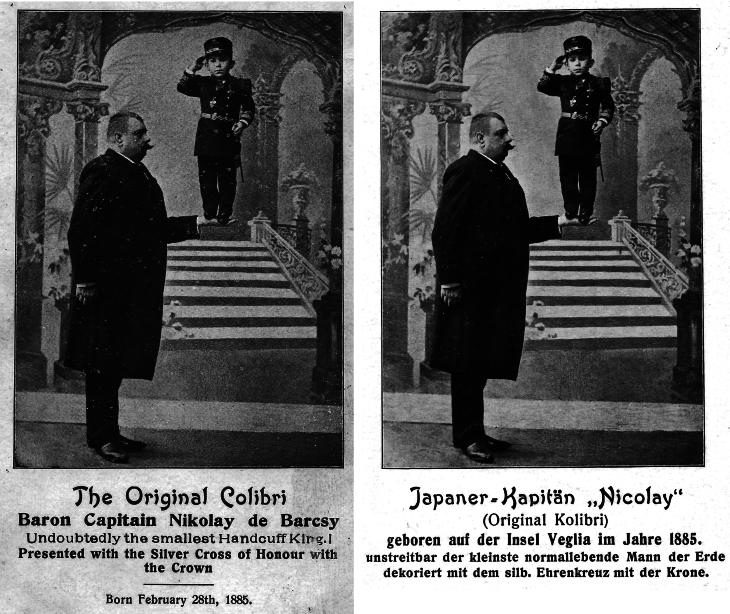
A and B. Nicu de Barcsy (28 February 1885 – 31 July 1976) standing on the hand of his father, Anton de Barcsy (1854–1952). Postcard in Figure 4B stating that he originated from the island of Krk (Veglia). Postcards in the collection of WW de Herder.

**Figure 5:**
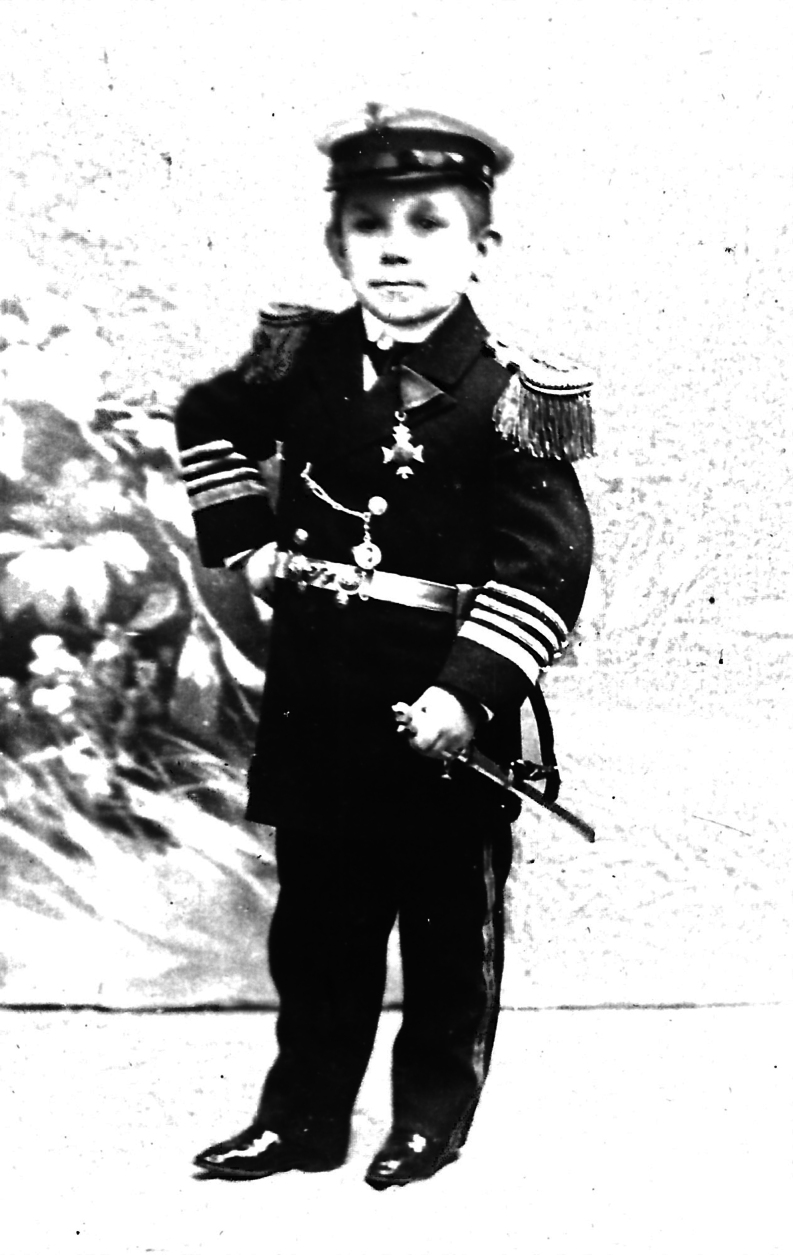
Nicu de Barcsy (28 February 1885 – 31 July 1976) dressed as Baron Capita(i)n. Fragment from a picture postcard. Postcard in the collection of WW de Herder.

Anton (more probably: Antal, born circa 1854) and Sidonia (more probably: Szidόnia) de Barcsy (more probably: Barcsay) were of Hungarian descent and aristocratic nobility. Baroness Sidonia was born on 1 May 1866. At the age of 18, on 28 February 1885, she gave birth to a son, who was named Nicu (more probably: Nicolaus) (1). While perfectly proportioned, Nicu was very small and weighed less than 1 kg (1 pound and 3 quarters). Within 2 weeks after his birth, Sidonia began to sprout a downy covering on her cheeks that rapidly grew. Soon a full-grown chestnut-colored beard covered the baroness’ cheeks (Figs. 1, 2, 3). Meanwhile, Nico still hardly grew. Baron Anton strongly opposed his wife shaving her beard. Almost simultaneously, a third problem arose as well: the baron had lost his fortune in poor investments, and around 1890, the family moved to Western Europe. Here, the penniless ‘de Barcsy Troupe’ found employment in the side shows of traveling circuses. Madame de Barcsy was advertised as the bearded lady and Nicu was billed as ‘The smallest Perfect Man on Earth’. The 1.90 m (6 ft 3 in) tall Baron weighted more than 180 kg (400 pounds) and sometimes performed as the circus’ strong man and Nicu used to dance on his outstretched hand (Fig. 4A/B). In 1903, when Nicu was 18 years old, the family immigrated to the USA. Nicu wore the Hungarian Silver Cross, which he claimed was his birthright, and wanted to be addressed as ‘Capita(i)n’ (Fig. 5). The family worked with many traveling shows, like the Hagenbeck and Wallace Show, the Ringling Brothers – Barnum and Bailey Circus, and the Campbell Brothers Circus. They would spend the winter in their New York apartment where they could live a luxurious life. However, in 1912, the baron died, aged 58. Soon, baroness Sidonia remarried a fellow trouper, a trick roper named Valentine Frederick ‘Fritz’ Tischer (1890–1951). Tischer claimed to be part Indian, part German, and was billed as ‘The Long-Haired Cherokee Buck Man’ and allegedly treated Sidonia and Nicu quite poorly. After Buck had sold all the fine furnishings of the New York apartment in 1914 to cover his gambling debts, the family was forced back into desperate circumstances. The trio found employment at the Campbell Brothers Circus again and finally settled in Drummond, Garfield County, Oklahoma, USA. Baron Nicu was advertised as ‘card tricks and performed on, or with small animals’. Baroness/madame Sidonia de Barcsy was diagnosed with diabetes and was not able to work as much in the side shows anymore. Finally, when she was not regularly bringing in a paycheck, Buck abandoned her and Nicu. Despite the fact that no divorce had ever been granted, Buck now lived with and later married Dolletta B Dodd (Boykin) (1881–1948), a widow of small stature who was also working in side shows billed as ‘the smallest mother in the world’, and fathered one child of normal stature by her. Dolletta Dodd already had two children of normal stature from an earlier marriage with ‘Major’ James Almerine Boykin, who was also of short stature. Sidonia de Barcsy never shaved her beard, which meanwhile had turned grey. Her statement was: ‘God sent the beard to me and I won’t take it off’. Once she went as far as she could by dyeing her beard blue and billing herself as ‘Lady Bluebeard’. She died, aged 59, on 19 October 1925 from a complicated severe cold in combination with diabetes mellitus. Her body was cremated in Kansas City MO and her ashes were sent to New York to rest in a receptacle beside those of her first husband Baron Anton de Barcsy. Nicu, or Nick as he also called himself, went on to perform at Coney Island, New York, as a magician and escape artist until he retired from the circus in 1932 at the age of 47. He was scared of becoming kidnapped, particularly by Cherokee Buck, and was convinced that the dust of the road would kill him. The exact height of Nick was not known, since he refused to be measured, but as an adult, he would stand only 90 cm (3 ft) tall. He never desired to return to Hungary nor to claim the family possessions there and at the age of 66, he became a naturalized citizen of the United States. In the 1960s he frequently became hospitalized for progressive kidney disease and finally had to move into a retirement center in Enid, Garfield County, Oklahoma, USA. He died in an Enid hospital on 31 July 1976 at the age of 91. He was buried at the Del Norte Cemetery southwest of Drummond under a marble monument marked ‘Baron Capitain Nicu de Barcsy, The Smallest Perfect Man on Earth’.

## Discussion

Nicu de Barcsy was a normal-proportioned person of short stature (less than 1 m). Idiopathic short stature can have different causes (2). Interestingly, a historic German postcard from that time mentions that he originated from Veglia, which is the Italian name for the Croatian island Krk (Fig. 4B). Due to isolation and consanguineous marriages, this island is well-known because of the increased prevalence of specific hereditary diseases like idiopathic small stature. The Hanhart's dwarfs on the island of Krk, as they have been called, have recessively inherited multiple pituitary deficiencies (MPHD) due to a mutation in a pituitary transcription factor gene, the *PROP1* gene (3, 4, 5, 6). With adequate hormonal substitution, these patients can live a long life and it should be noted that Nicu de Barcsy also became 91 years old (3). According to the available information, Nicu never received any hormonal treatment. Also, there is no indication that he originated from the island of Krk, nor that he was adopted from that area. Therefore, despite the fact that a relationship with the island Krk and its small stature people had been suggested, it is more probable that Nicu de Barcsy suffered from isolated GH deficiency only. In the case of multiple pituitary insufficiencies, it is difficult to explain how he could survive for so long without any hormonal replacement therapy. It is also not plausible that renal insufficiency was the main cause of the growth delay since reports only mention that he suffered from kidney disease later in life. Growth retardation is one of the sequelae of chronic kidney disease in young children, but it is highly improbable that Nicu de Barcsy could have survived for such a long time with childhood-onset chronic kidney disease.

Sidonia de Barcsy developed severe hirsutism. There is no evidence that she developed other phenomena of virilization. Hirsutism can have many causes, but since she survived for 41 years after the first diagnosis, malignant adrenal and ovarian tumors seem the most improbable causes. Also, she did not develop the full-blown phenotype of Cushing’s syndrome which excludes this diagnosis as well. The most probable causes of hirsutism in this case are luteoma of pregnancy, polycystic ovary syndrome (PCOS), or Sertoli–Leydig cell tumor(s) (7, 8, 9, 10, 11, 12). Since PCOS is closely linked to insulin resistance, the development of diabetes at a later age fits well into this diagnosis (13, 14, 15). In patients with luteoma of pregnancy, signs and symptoms of androgen excess usually present during pregnancy and these tend to disappear post-partum, but exceptions have been described (11). The fact that the hirsutism started immediately after pregnancy does not seem to fit with the diagnosis of luteoma of pregnancy (11). Although maternal virilization in PCOS during pregnancy has been described, postpartum virilization is also not typical of PCOS (7, 16). Late-onset (congenital) adrenal hyperplasia is another diagnosis which cannot be excluded. However, one would expect amenorrhea, decreased fertility and a relation between pregnancy and the development of hirsutism directly post-partum with this condition also cannot be easily explained (10). Androblastomas, or Sertoli–Leydig cell tumors are rare androgen-producing ovarian tumors that mostly occur between the ages of 20 and 40. The well-differentiated mostly unilateral tumors usually follow a benign course, but poorly differentiated malignant varieties which metastasize do also exist. These tumors have also been described in combination with pregnancy (12, 17).

The case of Sidonia de Barcsy matches with another famous historical case of hirsutism occurring shortly after pregnancy in Magdalena Ventura from Abruzzi, Italy, who developed a beard at the age of 37. She gave birth to seven children: three were born before the beard started to grow and four were born after she had grown the beard. In 1631, at the age of 52, she and her husband were pictured in a painting by the Spanish artist José de Ribera (1591–1652). In the painting, which is currently on display in the Prado Museum in Madrid, Spain, she is breastfeeding her most recently born child in 1631 (18, 19).

Bearded ladies were very popular actors in sideshows and traveling circuses in the 19th century and the beginning of the 20th century (20). Like the famous French bearded woman Clementine Delait, Sidonia de Barcsy was proud of her beard and did not feel the urge, nor the social pressure to shave and she was treated with dignity (20). The combination of severe post-partum hirsutism and offspring with short stature has not been described before and since a causal relationship is not evident this should be merely considered as coincidental. A relationship between virilization of the female fetus and hyperandrogenism in women has been described, however (10, 11, 17).

## Declaration of interest

The author declares that there is no conflict of interest that could be perceived as prejudicing the impartiality of the study reported.

## Funding

This work did not receive any specific grant from any funding agency in the public, commercial, or not-for-profit sector.

## Patient consent

Every effort was made to contact the next of kin of the deceased patient to obtain consent but was unsuccessful.

## Patient’s perspective

The female patient with the beard stated: ‘God sent the beard to me and I won’t take it off’.

## Author contribution statement

WW de Herder solely contributed to this article.
